# TSC1 deficiency drives immune evasion in colorectal cancer via mTORC1-mediated dysregulation of PD-L1 sialylation

**DOI:** 10.3389/fimmu.2025.1692210

**Published:** 2025-12-09

**Authors:** Xuemei Guan, Fengru Zhang, Yao Tang, Pengyu Bai, Wenyuan Wang

**Affiliations:** Shanxi Provincial Cancer Hospital, Taiyuan, China

**Keywords:** TSC1, mTORC1 signaling, sialylation, PD-L1 stability, tumor immune evasion, prognostic biomarker, colorectal cancer

## Abstract

**Background:**

TSC1 serves as a critical regulator of the mTORC1 signaling pathway with established roles in colorectal cancer pathogenesis. This investigation systematically examined the clinical relevance of TSC1 in colorectal cancer and its mechanistic relationship with sialylation-mediated immune regulation through integrated analysis of TCGA datasets and experimental validation.

**Methods:**

We employed bioinformatic analysis of TCGA cohorts combined with *in vitro* and *in vivo* experimental models. Molecular mechanisms were interrogated using biochemical assays, transcriptional profiling, and targeted pathway interventions. Sialylation dynamics were quantified through lectin-binding assays and surface plasmon resonance analysis.

**Results:**

Clinical analysis revealed that reduced TSC1 expression was significantly associated with poor prognosis in patients with colorectal cancer. Loss of TSC1 markedly activated the mTORC1 signaling pathway and induced upregulation of the sialyltransferase ST6GALNAC1 together with downregulation of the sialidase NEU4, thereby enhancing α2,6-sialylation on the cell surface. Treatment with rapamycin suppressed these alterations, whereas TSC1 knockdown partially reversed the inhibitory effects of rapamycin. This metabolic reprogramming led to increased α2,6-sialylation of PD-L1, which in turn elevated its protein stability and binding affinity to PD-1, ultimately resulting in T cell dysfunction and promoting tumor immune evasion. Both cellular and animal models demonstrated that pharmacological inhibition of mTORC1 or downregulation of ST6GALNAC1 effectively alleviated the aberrant PD-L1 glycosylation caused by TSC1 deficiency, thereby restoring the function of tumor-infiltrating CD8^+^ T cells and suppressing tumor progression.

**Conclusion:**

Our findings demonstrate that TSC1 deficiency promotes immune evasion through mTORC1-mediated reprogramming of PD-L1 glycosylation, particularly α2,6-sialylation. This study identifies TSC1 as a prognostic biomarker and defines the TSC1/mTORC1/glycosylation axis as a potential therapeutic target to improve immune suppression in colorectal cancer, providing fundamental insights for the development of precision immunotherapy strategies.

## 1 Introduction

Colorectal cancer (CRC) is the third most commonly diagnosed malignancy and the second leading cause of cancer-related death worldwide, with approximately 1.9 million new cases and 900,000 deaths reported annually (1–3). Despite advances in surgery, chemotherapy, and targeted therapies, the prognosis for advanced CRC remains unsatisfactory, largely due to tumor immune evasion and therapeutic resistance (4, 5). Although immune checkpoint blockade (ICB) therapy targeting PD-1/PD-L1 has shown remarkable efficacy in a subset of microsatellite instability-high (MSI-H) CRC patients, the majority of microsatellite-stable (MSS) tumors exhibit limited or no response (6, 7). These findings underscore the urgent need to elucidate the molecular mechanisms that drive immune evasion in CRC and to identify new regulatory pathways that can enhance immunotherapy responsiveness.

Recent advances in cancer glycobiology have revealed that post-translational modifications, particularly glycosylation, play a pivotal role in modulating immune checkpoint function (8, 9). PD-L1, a critical immune inhibitory ligand, undergoes extensive N-linked glycosylation, which protects it from proteasomal degradation and enhances its interaction with PD-1 on T cells (10, 11). Among various glycan structures, α2,6-linked sialic acid modifications have emerged as key determinants of PD-L1 stability and activity (12). Moreover, metabolic reprogramming in tumors, with particular emphasis on glucose and nucleotide metabolism, exerts a profound impact on sialylation by modifying sugar donor pools and enzymatic functions (13, 14). The interplay between glycosylation and metabolism is not only of great significance at the molecular level but may also influence clinical responses to immunotherapy. Moreover, with the expanding clinical use of PD-1 inhibitors, pharmacovigilance data have highlighted an urgent need to understand the molecular determinants underlying the safety and efficacy of immunotherapy (15). Despite growing recognition of this link, the upstream signaling pathways that orchestrate PD-L1 sialylation and metabolic control in CRC remain poorly defined.

The TSC1-mTORC1 axis serves as a central signaling hub integrating growth factors, nutrient availability, and metabolic cues to regulate cellular biosynthesis and immune homeostasis (16, 17). Recent multi-omics studies have identified metabolism-associated regulators as key modulators of tumor progression and immune evasion across multiple cancer types, further highlighting the link between metabolic signaling and immune remodeling (18). TSC1 (hamartin) functions as a key negative regulator of mTORC1, and its loss leads to sustained mTORC1 activation, metabolic reprogramming, and dysregulated protein translation (16, 19). Accumulating evidence indicates that mTORC1 activation in tumor cells not only drives anabolic metabolism but also reshapes the tumor immune microenvironment, affecting antigen presentation, cytokine production, and T-cell infiltration (20–22). Intriguingly, recent studies suggest that mTORC1 signaling may also influence glycosylation pathways, though the precise mechanisms remain to be elucidated (23). In CRC, TSC1 frequently exhibits genomic loss or transcriptional downregulation, correlating with advanced disease, poor prognosis, and reduced CD8^+^ T-cell infiltration (24, 25). These findings suggest that TSC1 may exert noncanonical functions beyond classical mTORC1 regulation, potentially linking metabolic signaling to immune modulation through glycosylation control.

Based on these observations, we hypothesized that TSC1 deficiency promotes tumor immune evasion in CRC through mTORC1-mediated dysregulation of PD-L1 sialylation. To test this, we systematically investigated how TSC1 loss affects the expression of key sialylation-related enzymes, including ST6GALNAC1, NEU4, and NPL, and examined the resulting impact on PD-L1 modification, stability, and immune suppression. This study identifies a previously unrecognized TSC1/mTORC1-sialylation axis that integrates metabolic regulation and immune checkpoint control, providing mechanistic insights into CRC immune evasion and potential therapeutic avenues for overcoming immunotherapy resistance.

Although accumulating evidence has linked mTOR signaling to tumor immunometabolism, how TSC1 modulates glycosylation-dependent immune evasion in colorectal cancer remains largely unknown. To address this gap, we systematically investigated the biological and clinical significance of TSC1, focusing on its mechanistic role in shaping the tumor immune microenvironment through aberrant sialylation. Through an integrated strategy involving clinical data, molecular and biochemical analyses, and immunological evaluation, we explored how the loss of TSC1 impacts the expression of sialylation-associated enzymes, with a focus on ST6GALNAC1, and how this modulation affects PD-L1 glycosylation, structural stability, and immunoregulatory function. Our study uncovers a previously unrecognized TSC1-mTORC1-sialylation axis that connects metabolic signaling to immune checkpoint regulation, revealing how loss of TSC1 drives immune escape through enhanced α2,6-linked sialylation. These findings identify TSC1 as both a prognostic biomarker and a mechanistic bridge between metabolism and immune regulation, providing a conceptual and experimental foundation for the rational design of mTOR-targeted and immune checkpoint–based combination therapies in colorectal cancer.

## 2 Materials and methods

### 2.1 Data acquisition and bioinformatics analysis

Gene expression profiles and corresponding clinical data, including age, gender, tumor stage, and survival information, were obtained from the TCGA-COAD dataset through the UCSC Xena browser (https://xenabrowser.net/datapages/). Differential gene expression analysis was performed between 471 tumor samples and 41 normal colon tissues using the DESeq2 package (version 1.30.1) in R, with genes showing absolute log2 fold change > 1 and false discovery rate (FDR) adjusted p-value < 0.05 considered statistically significant. The normalized gene expression matrix was subjected to subsequent analyses, including immune cell infiltration estimation and survival correlation studies.

### 2.2 Immune infiltration analysis

The relative proportions of 22 immune cell subtypes were quantified using the CIBERSORT deconvolution algorithm with the LM22 signature matrix (1000 permutations, default parameters). To identify significant differences in immune cell infiltration between tumor and normal tissues, we performed Wilcoxon rank-sum tests with Benjamini-Hochberg correction for multiple comparisons (FDR < 0.05). Subsequently, Pearson correlation analysis was conducted to evaluate linear associations between TSC1 expression levels (log2-transformed TPM values) and immune cell infiltration fractions, with correlation coefficients (r) and corresponding p-values calculated for each immune subset.

### 2.3 Survival analysis

To systematically evaluate the prognostic significance of sialylation-related genes, we first performed univariate Cox proportional hazards regression analysis on 72 sialylation-associated genes obtained from the MSigDB database (Liberzon et al., 2015). Genes meeting the significance threshold (p < 0.05 and hazard ratio [Vrints, #39] ≠ 1) were subsequently incorporated into a multivariate Cox regression model to calculate risk scores for each patient. The risk score was computed as the linear combination of gene expression levels weighted by their respective regression coefficients. Patients were then stratified into high- and low-risk groups based on the median risk score cutoff.

The prognostic performance of the risk model was comprehensively assessed using three complementary approaches: (1) Kaplan-Meier survival curves with log-rank tests to compare overall survival between risk groups; (2) time-dependent receiver operating characteristic (ROC) analysis at 3-, 5-, and 7-year intervals to evaluate model discrimination accuracy; and (3) multivariate Cox regression adjusting for clinical covariates (age, gender, and tumor stage) to assess the independent prognostic value of the risk score.

### 2.4 Cell culture and genetic manipulation

Murine colorectal cancer cells (MC38, Kerafast, Cat# ENH204-FP) and normal colonic epithelial cells (YAMC, Kerafast, Cat# ENH282-FP) were maintained in DMEM/RPMI-1640 medium supplemented with 10% fetal bovine serum (FBS; Gibco, Cat# 10099141C) under standard culture conditions (37°C, 5% CO_2_, humidified atmosphere). All cell lines were routinely authenticated by short tandem repeat (STR) profiling and tested for mycoplasma contamination monthly.

For genetic manipulation, TSC1 knockdown was achieved through lentiviral transduction using validated shRNA constructs (MISSION^®^ shRNA, Sigma-Aldrich, Cat# SHCLNG-NM_000368), with transduction efficiency optimized via polybrene treatment (Sigma-Aldrich, Cat# TR-1003-G; 8 μg/mL). Successful knockdown was confirmed by both quantitative real-time PCR (qRT-PCR; ≥70% mRNA reduction) and Western blot analysis (≥80% protein reduction) compared to non-targeting shRNA controls (MISSION^®^ Non-target shRNA Control, Sigma-Aldrich, Cat# SHC016). For functional rescue experiments, TSC1-deficient cells were transfected with pCMV6-ST6GALNAC1 expression vector (OriGene, Cat# RC201964) using Lipofectamine 3000 (Thermo Fisher Scientific, Cat# L3000008), with empty vector-transfected cells serving as controls. All genetic modifications were validated at both transcriptional (qRT-PCR) and translational (Western blot) levels before functional assays. The sequences of all shRNA constructs used for TSC1 silencing and the corresponding control are summarized in **Supplementary Table S3**.

### 2.5 Transcriptomic and single-cell sequencing analysis

For transcriptomic analysis, RNA sequencing (RNA-seq) libraries were prepared using the Illumina TruSeq RNA Sample Preparation Kit (Illumina, USA) and sequenced on an Illumina NovaSeq 6000 platform. The sequencing data were processed using standard bioinformatics pipelines, including quality control, read alignment, and differential expression analysis.

The single-cell data are from the dataset GSE261388. Sequence alignment was accomplished through cellranger 7.0, data reading was performed using the Seurat package, basic QC (outlier 5%) was completed, double-cell filtering was carried out using scDblFinder, and batch effects were removed with harmony. Cell annotation uses FindClusters in the Seurat package for clustering at a resolution of 0.1. The FindAlIMarkers in the Seurat package (logfc.threshold = 0.1, min.pct = 0.01, adjusted P value <0.05) were used to identify the marker genes of each cluster and perform cell type annotation. Use clusterprofile for kegg go annotation. To better monitor the status of sialic acid-related genes among the samples, pseudo-bulk analysis was constructed. The sialylation-related gene set was selected from the MsigDB database for gene analysis, and it was found that ST6GALNAC1 was highly expressed in the tumor group. Gene association analysis using the cor package revealed that genes such as ST6, GalNAC1, B3GNT7, MEU4, B3NT3, and CHST5 had relatively high correlations.

### 2.6 Gene expression analysis

The transcriptomic data were analyzed using the DESeq2 package to identify differentially expressed genes (DEGs) between the TSC1_ShRNA knockdown cells and control cells. Gene ontology (GO) and pathway enrichment analysis were performed using the DAVID Bioinformatics Resources to explore the biological functions of these genes. Heatmaps of gene expression were generated using the pheatmap R package.

In single-cell analysis, gene expression patterns were visualized and clustered using Uniform Manifold Approximation and Projection (UMAP) to assess the heterogeneity of gene expression in different cell populations. Co-expression modules were identified using the WGCNA package, and gene correlation analysis was performed to explore gene-gene interactions within different cell types.

### 2.7 Functional enrichment and pathway analysis

Functional enrichment analysis was conducted to investigate the biological processes and pathways associated with differentially expressed genes. KEGG pathway analysis was performed using the clusterProfiler R package to identify pathways involved in immune response, antigen processing, and other tumor-related processes. Gene sets related to immune regulation and inflammation were prioritized based on their relevance to tumor progression.

### 2.8 Wound healing assay

The wound healing assay was performed to evaluate the migratory and proliferative capacities of TSC1-knockdown (KD) versus control (Control) HCT116 colorectal cancer cells. Cells were seeded in 6-well plates (Corning) and cultured in DMEM high glucose medium (Gibco) supplemented with 10% fetal bovine serum (FBS; HyClone) until reaching >90% confluence. Prior to scratching, the monolayer was washed twice with sterile PBS (1×, pH 7.4) to remove non-adherent cells. Uniform linear wounds were created using a 200 μL sterile pipette tip (Greiner Bio-One) held perpendicular to the plate surface, with consistent pressure applied across all wells. Following two additional PBS washes to remove cellular debris, the medium was replaced with low-serum (2% FBS) DMEM to minimize proliferation-driven wound closure while permitting migration-mediated healing.

For quantitative analysis, phase-contrast images of wound areas were captured at 0, 24, and 48 h post-scratching using an inverted fluorescence microscope (Olympus IX83) equipped with a 10× objective and maintained at 37°C/5% CO_2_. Three representative fields per well were imaged at each timepoint, with consistent positioning ensured by a mechanical stage. Wound widths were measured using ImageJ software (NIH, v1.53) with the MRI Wound Healing Tool plugin, applying the following analytical parameters: (1) manual selection of wound edges, (2) threshold-based area calculation, and (3) normalization to initial wound area (0 h).

### 2.9 Transwell migration assay

The Transwell chamber system (Corning, 8 μm pore size) was employed to quantitatively assess the effect of TSC1 knockdown on tumor cell migration. Chambers were pre-coated with Matrigel basement membrane matrix (BD Biosciences) diluted 1:8 in ice-cold serum-free medium and polymerized at 37°C for 1 h to simulate the extracellular matrix barrier. After removing excess liquid, chambers were equilibrated with serum-free medium for 30 min at 37°C.

For migration assays, TSC1-knockdown and control cells were trypsinized, washed with PBS, and resuspended in serum-free medium at a density of 5×10^5^ cells/mL. Cell suspensions (200 μL) were carefully added to the upper chambers, while the lower chambers contained 600 μL of complete medium with 10% FBS as a chemoattractant. Following 24 h incubation (37°C, 5% CO_2_), non-migrated cells on the upper membrane surface were removed using cotton swabs. Migrated cells on the lower surface were fixed with methanol (15 min), air-dried, and stained with 0.1% crystal violet (20 min). After PBS washing, five random fields per membrane were imaged under phase-contrast microscopy (100× magnification), and migrated cells were quantified using ImageJ software with particle analysis function.

### 2.10 Histological immunofluorescence staining

Formalin-fixed, paraffin-embedded (FFPE) colorectal tissue sections were obtained from the Shanxi Provincial Cancer Hospital. All tissue samples used in this study were archival materials collected for diagnostic purposes and provided anonymously by the pathology department (approval No. 2023GZR03). For immunofluorescence staining, 4-μm-thick sections were deparaffinized in xylene and rehydrated through a graded ethanol series (100%, 95%, 80%, and 70%). Antigen retrieval was performed in citrate buffer (10 mM, pH 6.0) using a microwave oven for 10 min, followed by cooling to room temperature. Sections were then permeabilized with 0.3% Triton X-100 in PBS for 10 min and blocked with 5% bovine serum albumin (BSA; Sigma-Aldrich, Cat# A7906) for 1 h at room temperature. Tissue sections were incubated overnight at 4 °C with the following primary antibodies: anti-TSC1 rabbit monoclonal antibody (1:200, Cell Signaling Technology, 6935S), anti-PD-L1 rabbit monoclonal antibody (1:200, Cell Signaling Technology, 13684S), anti-ST6GALNAC1 goat polyclonal antibody (1:100, Invitrogen, PA5-31200), and biotinylated Sambucus Nigra Lectin (SNA, 1:500, Vector Laboratories, B-1305-2) to detect α-2,6 sialylated glycoconjugates. After three washes with PBS, fluorescently labeled secondary antibodies (Alexa Fluor 488 anti-rabbit, 1:500; Alexa Fluor 594 anti-goat, 1:500) and Streptavidin-Alexa Fluor 647 (1:500) were applied. Nuclei were visualized with DAPI. Finally, slides were mounted using Fluoromount-G™ Mounting Medium (SouthernBiotech, Cat# 0100-01) and imaged under a Leica TCS SP8 confocal microscope. Images were processed and analyzed using ImageJ to quantify fluorescence intensity and cellular localization.

### 2.11 Quantitative real-time PCR analysis

Total RNA was isolated from tissue samples using TRIzol reagent (Invitrogen, Thermo Fisher Scientific, Cat# 15596026) following the manufacturer’s protocol, with RNA integrity verified by 1% agarose gel electrophoresis and purity assessed by NanoDrop spectrophotometry (A260/A280 ratio >1.8). Genomic DNA contamination was eliminated using the gDNA Clean Reagent from the Evo M-MLV Reverse Transcription Kit (Accurate Biotechnology, Cat# AG11728), followed by first-strand cDNA synthesis with oligo(dT)18 and random hexamer primers in a 20 μL reaction volume under the following conditions: 42°C for 15 min and 85 °C for 5 sec. For qPCR amplification, reactions were performed in triplicate using the SYBR Green Pro Taq HS Premix (Accurate Biotechnology, Cat# AG11701)) on a QuantStudio 6 Flex Real-Time PCR System (Applied Biosystems, Thermo Fisher Scientific). Each 20 μL reaction contained 1 μL cDNA template, 0.4 μM gene-specific primers, and 10 μL 2× SYBR Green master mix. The thermal cycling protocol consisted of: (1) initial denaturation at 95°C for 30 sec; (2) 40 cycles of 95°C for 5 sec and 60°C for 30 sec; (3) melt curve analysis from 65°C to 95°C (0.5 °C increments). Primer specificity was confirmed by single peak melt curves and agarose gel verification of amplicon size. Relative gene expression was calculated via the 2-ΔΔCt method using GAPDH as the endogenous control, with inter-assay variability controlled by including three technical replicates and two biological replicates per sample. The primer sequences used in this study are listed in **Supplementary Table S2**.

### 2.12 Western blot analysis

Tissue samples were homogenized in RIPA lysis buffer supplemented with 1 mM PMSF using a mechanical homogenizer, followed by centrifugation at 12,000 g for 15 min at 4°C. The supernatant was collected and stored at -80 °C. Protein concentration was determined by BCA assay (Pierce) according to the manufacturer’s protocol, using bovine serum albumin as standard. Samples were normalized to equal concentrations, mixed with 5× Laemmli buffer, and denatured at 95°C for 10 min. Proteins (20-30 μg/lane) were separated on 10% SDS-PAGE gels (5% stacking gel) at 80 V for 30 min followed by 120 V for 60–90 min. Proteins were transferred to methanol-activated PVDF membranes (Millipore) using wet transfer system at 100 V for 90 min. Membranes were blocked with 5% non-fat milk in TBST for 1 h at room temperature, then incubated with primary antibodies (diluted in blocking buffer) overnight at 4°C. The following primary antibodies were used for immunoblotting: anti-NEU4 rabbit monoclonal antibody (1:1000, Invitrogen, PA5-57936), anti-PD-L1 mouse monoclonal antibody (1:2000, Cell Signaling Technology, 13684S), anti-ST6GALNAC1 goat polyclonal antibody (1:500, Invitrogen, PA5-31200), and anti-β-tubulin rabbit monoclonal antibody (1:5000, ABclonal, A12289). After three TBST washes (10 min each), membranes were incubated with HRP-conjugated secondary antibodies for 1 h at room temperature. Protein bands were visualized using enhanced chemiluminescence substrate (ECL, Thermo Scientific) and imaged with a chemiluminescence detection system. Band intensities were quantified using ImageJ software (NIH), normalized to β-tubulin loading control, and expressed as relative optical density units.

### 2.13 Sialylation analysis by lectin blotting

For sialylation profiling, tumor cells from experimental groups were lysed in RIPA buffer supplemented with protease inhibitors, and total protein concentrations were determined using the BCA assay. Equal amounts of protein (30 μg per lane) were resolved on 4-20% gradient SDS-PAGE gels (Bio-Rad) and electrophoretically transferred to PVDF membranes using a wet transfer system (25 V, 4°C, 16 h). Membranes were blocked with 5% non-fat dry milk in TBST for 1 h at room temperature before incubation with biotinylated Sambucus nigra lectin (SNA, Vector Laboratories; Cat# B-1305; 1:1000 dilution in blocking buffer) at 4°C overnight to specifically detect α2,6-linked sialic acid modifications.

Following three washes with TBST (10 min each), membranes were probed with horseradish peroxidase (HRP)-conjugated streptavidin (Vector Laboratories, Cat# SA-5004; 1:5000 dilution in TBST) for 1 h at room temperature. Signal detection was performed using enhanced chemiluminescence (ECL Prime, GE Healthcare) and quantified using a chemiluminescence imaging system (Bio-Rad ChemiDoc MP).

To normalize sialylation levels to total PD-L1 protein expression, parallel Western blot analysis was conducted using anti-PD-L1 primary antibody (Cell Signaling Technology, #13684; 1:1000) and HRP-conjugated secondary antibody under identical electrophoretic conditions. Band intensities were analyzed using Image Lab software (v6.0), with sialylation levels expressed as the ratio of SNA signal to corresponding PD-L1 signal for each sample.

### 2.14 CD8+ T cell-mediated cytotoxicity assay

The cytotoxic activity of CD8+ T cells against tumor cells was evaluated using a calcein-AM release assay combined with Annexin V/PI apoptosis detection. Tumor cells (HCT116 wild-type and TSC1-knockdown) were labeled with 5 μM calcein-AM (Thermo Fisher Scientific) for 30 min at 37°C, then co-cultured with allogeneic CD8+ T cells (isolated from healthy donor PBMCs using MACS separation, Miltenyi Biotec) at 10:1 effector-to-target ratio in RPMI 1640 medium supplemented with 20 ng/mL recombinant human IL-2 (PeproTech). After 24 h co-culture, cells were harvested using EDTA-free trypsin and stained with Annexin V-FITC/propidium iodide (PI) apoptosis detection kit (BD Biosciences) according to manufacturer’s protocol. Flow cytometry analysis was performed on a BD FACS Canto II system, with tumor cell populations gated based on calcein-AM fluorescence and apoptotic cells identified as Annexin V+/PI+ (late apoptosis) or Annexin V+/PI- (early apoptosis). To assess PD-L1 sialylation-dependent immune evasion, parallel experiments included anti-PD-L1 blocking antibody (10 μg/mL, clone 29E.2A3, BioLegend) treatment groups. Spontaneous release controls (tumor cells alone) and maximum release controls (tumor cells lysed with 1% Triton X-100) were included for assay validation. Specific cytotoxicity was calculated as: [(experimental release - spontaneous release)/(maximum release - spontaneous release)] × 100%.

### 2.15 *In vivo* tumorigenesis and therapeutic evaluation

Experimental C57BL/6J mice were purchased from Guangdong Laboratory Animal Monitoring Institute. All animal experiments were approved by Laboratory Animal Management and Ethics Committee of Guangdong Laidi Biomedical Research Institute Co., LTD (Ethics Approval No. 2025025-1]), and were conducted in strict accordance with animal welfare and ethical guidelines. The experimental animals were housed in an SPF-grade environment, with temperature maintained at 22 ± 2 °C, relative humidity at 50–60%, and a light/dark cycle of 12 hours/12 hours (light period: 7:00–19:00). Mice were kept in individually ventilated cages (IVC). Bedding, feed, and drinking water were sterilized by high-pressure steam before use, and bedding was changed twice a week. TSC1-knockdown MC38 cells and control MC38 cells were prepared as single-cell suspensions, adjusted to a density of 5×10^7^ cells/mL with PBS, and then mixed with pre-cooled Matrigel at a 1:1 volume ratio (resulting in a final cell density of 2.5×10^7^ cells/mL; each inoculation site received 100 μL containing 5×10^6^ cells). All procedures prior to inoculation were performed on ice to prevent Matrigel solidification.

Six-week-old C57BL/6J wild-type mice were selected for subcutaneous inoculation under a biosafety cabinet. After anesthesia via isoflurane inhalation, the central area of the right dorsal region was disinfected with 75% alcohol. A 1 mL sterile syringe was used to aspirate 100 μL of the cell-Matrigel mixture, which was slowly injected subcutaneously to form a uniformly raised inoculation site. After inoculation, the mice were monitored until full recovery and returned to their housing cages upon confirmation of normal condition. Starting from the second day after inoculation, the general condition and tumor formation of the mice were monitored daily. Tumor length (L) and width (W) were measured every other day using a vernier caliper, and tumor volume was calculated using the formula V = L × W² × 0.5. Once the tumor volume reached approximately 100 mm³, treatment groups were established according to the experimental design, and corresponding interventions were initiated.

Mice were randomized into four treatment groups (n=3 per group).: (1) anti-PD-1 monoclonal antibody (10 mg/kg, Bio X Cell, clone RMP1-14) administered intraperitoneally twice weekly; (2) rapamycin (2 mg/kg, LC Laboratories) delivered daily via oral gavage; (3) combination therapy (anti-PD-1 + rapamycin); and (4) vehicle control (PBS). Tumor dimensions were measured every three days using digital calipers, and body weights were recorded for toxicity assessment. At the experimental endpoint (when tumor volume ≥ 1500 mm³ or on day 28 after inoculation, whichever occurred first), euthanasia was performed on the mice to excise the tumors. The specific euthanasia procedure was as follows: mice were transferred to a dedicated euthanasia chamber, and compressed CO_2_ gas was introduced at a flow rate controlled to fill 30%–50% of the chamber volume per minute. Exposure continued until complete cessation of breathing (approximately 5 minutes). The mice were then removed, and cervical dislocation was performed as a secondary confirmation to ensure death. All procedures were carried out by personnel certified in laboratory animal euthanasia and strictly adhered to animal welfare and ethical guidelines. Excised tumors were: (1) weighed for gross evaluation, (2) divided for parallel processing - one portion fixed in 10% neutral buffered formalin for hematoxylin and eosin (H&E) staining and histopathological examination, and another portion dissociated into single-cell suspensions for immune profiling. Tumor-infiltrating lymphocytes were isolated using a mechanical dissociation protocol followed by Percoll gradient centrifugation. Cells were stained with fluorescent-conjugated antibodies against CD3 (clone 17A2), CD8 (clone 53-6.7), and FOXP3 (clone MF-14) for flow cytometric quantification (BD LSRFortessa). Data were analyzed using FlowJo software (v10.8.1) with gating strategies based on fluorescence-minus-one controls.

### 2.16 Statistical analysis

All statistical analyses were performed using R statistical software (version 4.0.3) and GraphPad Prism (version 9.0.0) with appropriate validation of test assumptions. Continuous variables with normal distribution (assessed by Shapiro-Wilk test and Q-Q plots) were analyzed using two-tailed Student’s t-test (for two-group comparisons) or one-way ANOVA with Tukey’s *post hoc* test (for multiple groups). Non-normally distributed data were evaluated using Mann-Whitney U test (two groups) or Kruskal-Wallis test with Dunn’s multiple comparisons (multiple groups). Survival curves were compared using log-rank tests with Kaplan-Meier estimation. Correlation analyses between gene expression and immune cell infiltration were conducted using Pearson’s correlation for linear relationships or Spearman’s rank correlation for monotonic non-linear associations. Multiple testing correction was applied using the Benjamini-Hochberg false discovery rate (FDR) method when appropriate. All statistical tests were two-sided, with p-values <0.05 considered statistically significant. Effect sizes were reported as mean differences (95% confidence intervals) for parametric tests, median differences for non-parametric tests, and hazard ratios for survival analyses. Graphical representations include box plots (median ± interquartile range), bar graphs (mean ± SEM), and forest plots for multivariate analyses.

## 3 Results

### 3.1 Sialylation levels are associated with prognosis in colorectal cancer

**Figure 1:**
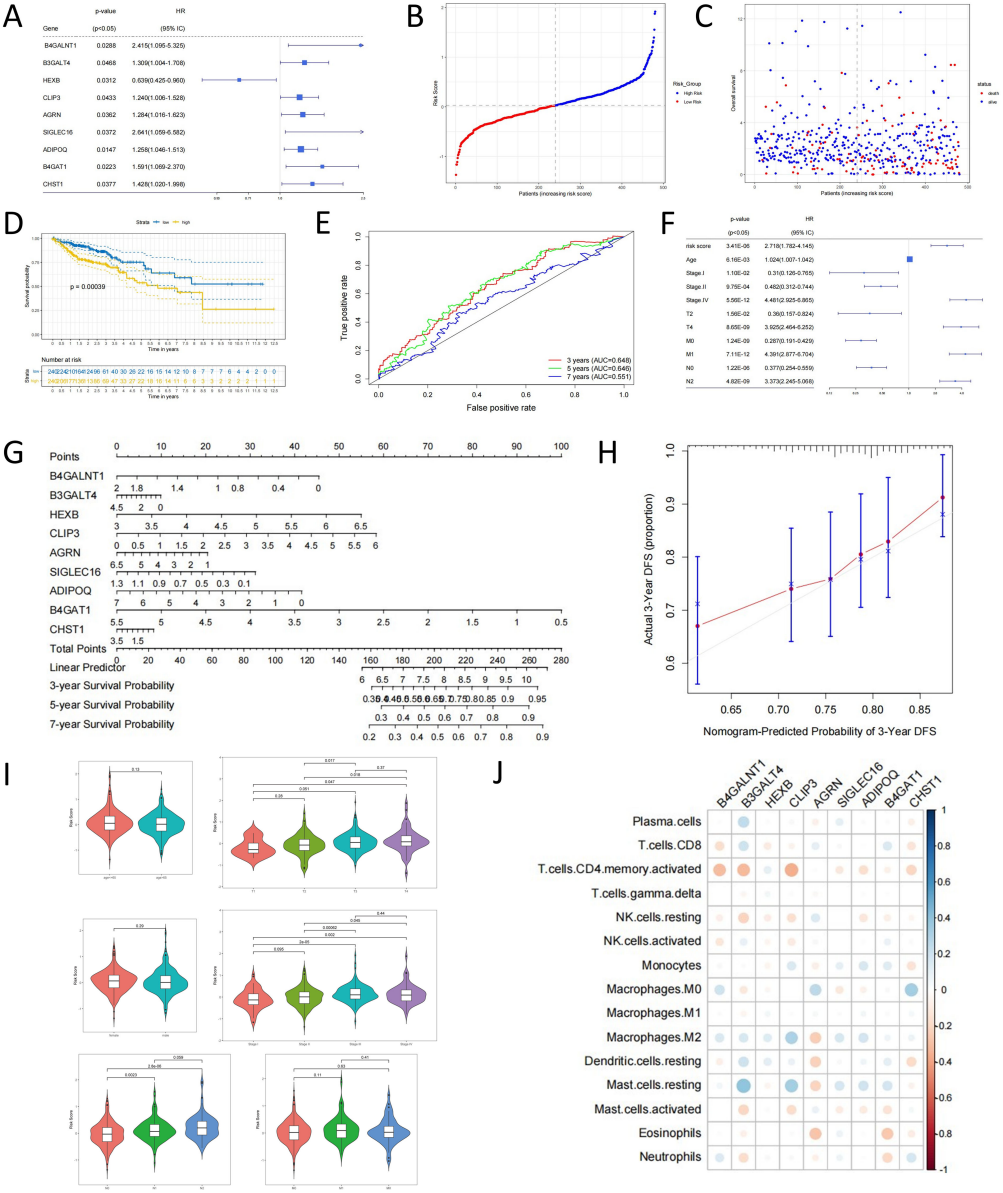
Analysis of the correlation between sialylation levels and tumor prognosis in colorectal cancer. **(A)** Prognostic impact of sialylation-associated genes by univariate Cox regression analysis. **(B)** Risk Score Distribution Plot. **(C)** Patient Survival Status Plot. **(D)** Kaplan-Meier analysis comparing survival outcomes across risk strata. **(E)** ROC curve analysis was performed to assess model efficacy by calculating the AUC. **(F)** Prognostic impact of risk score and clinical variables by univariate Cox regression. **(G)** Gene-based prognostic prediction model visualization. **(H)** Model calibration plot. **(I)** Association of risk stratification with clinicopathological features. **(J)** Association of immune cell infiltration patterns with prognostic gene signatures.

This study retrieved 72 sialylation-related genes from the MsigDB database and performed univariate Cox regression analysis (p<0.05 and HR≠1) to identify 9 genes significantly associated with overall survival (OS) in colorectal cancer patients. Among these, HEXB was identified as a protective factor, while the remaining 8 genes were associated with higher risk (**Figure 1A**). These results suggest that sialylation-related genes may play a crucial role in the biological behaviors of colorectal cancer, particularly in tumorigenesis, progression, and immune evasion.

To construct a risk scoring model, we performed multivariate Cox regression analysis on the 9 survival-related genes. By applying the risk scoring formula, we calculated the risk score for each patient and classified them into high-risk and low-risk groups based on the median risk score. The distribution of patients’ risk scores and survival status is shown in **Figures 1B, C**. To evaluate the prognostic value of the risk score model, we plotted the survival curves for high-risk and low-risk groups (**Figure 1D**). The survival analysis revealed a significant difference in overall survival between the two groups (p = 0.00039), with the high-risk group showing notably lower survival rates compared to the low-risk group. These findings further support the potential prognostic value of sialylation-related genes in colorectal cancer, suggesting that these genes could serve as important molecular biomarkers for predicting the survival of colorectal cancer patients.

To further validate the accuracy of the risk model, we used Receiver Operating Characteristic (ROC) curves to assess its performance. The ROC curve illustrates the model’s performance at various classification thresholds, with the Area Under the Curve (AUC) representing the prediction accuracy and sensitivity. A higher AUC value indicates better predictive accuracy. **Figure 1E** shows the ROC curves for 3-year, 5-year, and 7-year survival periods, with AUC values greater than 0.5 for all time points, indicating that the risk model has good predictive power. Notably, the improved AUC values suggest that the model can provide more accurate survival predictions for patients at different time points, highlighting its significant clinical relevance.

To determine whether the risk score is an independent prognostic factor beyond other clinical features, we performed univariate Cox regression analysis for both the risk score and clinical characteristics (e.g., age, gender, clinical stage, T-stage, M-stage, and N-stage) (**Figure 1F**). The results showed that the risk score remains an independent prognostic factor even after adjusting for other clinical characteristics. This indicates that the risk model based on sialylation-related genes can not only reflect the biological properties of tumors but also independently predict patient survival outcomes, showing strong clinical application potential.

To further optimize survival prediction, we constructed a nomogram model based on the expression levels of the prognostic genes. The nomogram (**Figure 1G**) illustrates the contribution of each variable to patient prognosis. Each variable is assigned a score based on its expression level, and the total score is calculated as the sum of all individual scores. This approach allows for a more intuitive assessment of each gene’s impact on prognosis and provides clinicians with a simplified predictive tool. To evaluate the accuracy of the nomogram, we plotted a calibration curve using the “regplot” package (**Figure 1H**). The closer the calibration curve is to the reference line (slope = 1), the higher the prediction accuracy of the model, providing strong evidence of the nomogram’s reliability for clinical use.

To explore the correlation between risk scores and clinical characteristics, we grouped patients from the TCGA dataset based on different clinical features and used the Wilcoxon rank-sum test to compare risk scores across different clinical subgroups (p<0.05). The results showed significant differences in risk scores between clinical subgroups (**Figure 1I**), further confirming the clinical applicability of the risk score model. In particular, risk scores were found to provide valuable prognostic information for patients with different clinical stages, T-stage, M-stage, and N-stage.

To investigate the relationship between immune cell infiltration and prognostic genes, we performed Pearson correlation analysis and generated a correlation heatmap (**Figure 1J**). The analysis revealed that resting mast cells were highly positively correlated with other resting mast cells (r = 0.393, p = 2.40E-20), M2 macrophages showed a positive correlation with the CLIP3 gene (r = 0.301, p = 3.75E-12), and M0 macrophages were positively correlated with the CHST1 gene (r = 0.335, p = 3.35E-12). These results suggest that sialylation-related genes may influence immune evasion and tumor microenvironment remodeling by regulating immune cell infiltration and function, providing new insights into the role of these genes in immune regulation.

In conclusion, this study constructed a risk score model based on sialylation-related genes to assess the survival prognosis of colorectal cancer patients and explored the correlation between immune cell infiltration and prognostic genes. Our findings not only validate the prognostic value of sialylation-related genes in colorectal cancer but also provide important clues for future research into the role of these genes in tumor immune microenvironments.

### 3.2 TSC1 expression is associated with immune cell infiltration patterns in colorectal cancer

**Figure 2:**
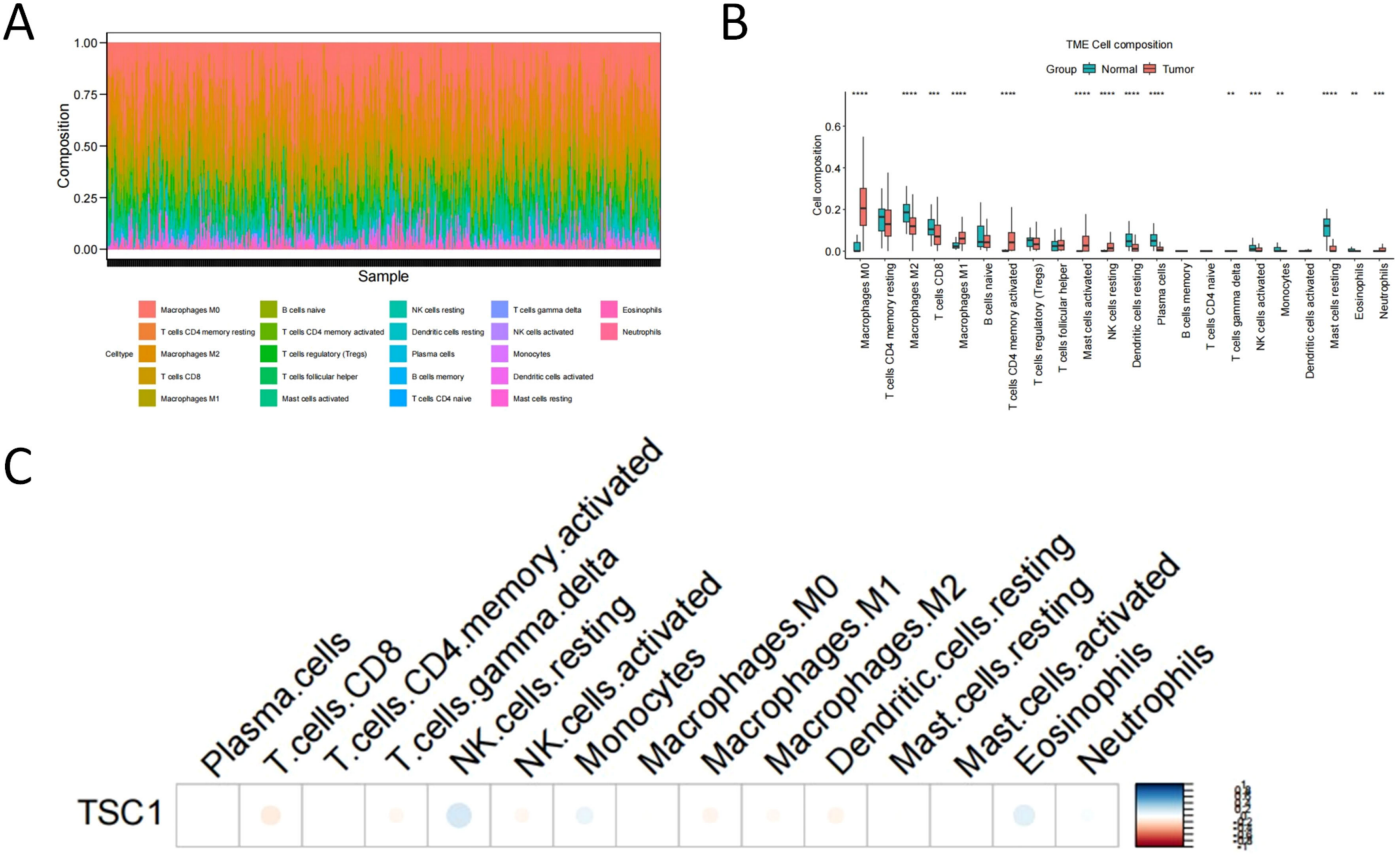
Immune infiltration analysis of TSC1 in colorectal cancer. **(A)** Immune Infiltration Landscape. **(B)** Comparative analysis of immune infiltration across experimental groups. **(C)** Association of TSC1 expression with distinct immune cell populations.

In this study, we obtained gene expression profiles and clinical characteristics (including Age, Gender, Subtype, Grade, Stage, etc.) of the TCGA-COAD dataset from the UCSC Xena database (https://xenabrowser.net/datapages/). A total of 512 samples were included, comprising 471 tumor tissues and 41 normal tissues. To analyze differences in the immune microenvironment between tumor and normal tissues, we employed the CIBERSORT algorithm to calculate the infiltration abundance of 22 immune cell types (**Figure 2A**).

Wilcoxon rank-sum test results revealed significant differences in infiltration levels of 15 immune cell types between tumor and normal control groups (**Figure 2B**), including: Macrophages M0, Macrophages M2, T cells CD8, Macrophages M1, T cells CD4 memory activated, Mast cells activated, NK cells resting, Dendritic cells resting, Plasma cells, T cells gamma delta, NK cells activated, Monocytes, Mast cells resting, Eosinophils, and Neutrophils.

Furthermore, we investigated the relationship between TSC1 expression and differential immune cell infiltration. Pearson correlation analysis demonstrated that TSC1 expression showed significant positive correlations with NK cells resting, Eosinophils, and Monocytes, while exhibiting a significant negative correlation with T cells CD8 (**Table 1**, **Figure 2C**). These results suggest that TSC1 may play an important role in regulating the immune microenvironment of colon cancer.

**Table 1:** Association of TSC1 expression with immune cell infiltration patterns using Pearson correlation.

Patterns	*p*	r
NK.cells.resting	0.00003	0.18336
Eosinophils	0.00224	0.13481
T.cells.CD8	0.00973	-0.11416
Monocytes	0.04070	0.09048

### 3.3 Sialic acid-related gene expression patterns are associated with tumor phenotypes at bulk and single-cell transcriptomic levels

Our research results support the key role of sialic acid-related genes in regulating various biological processes, including immune regulation and tumor progression.

**Figure 3:**
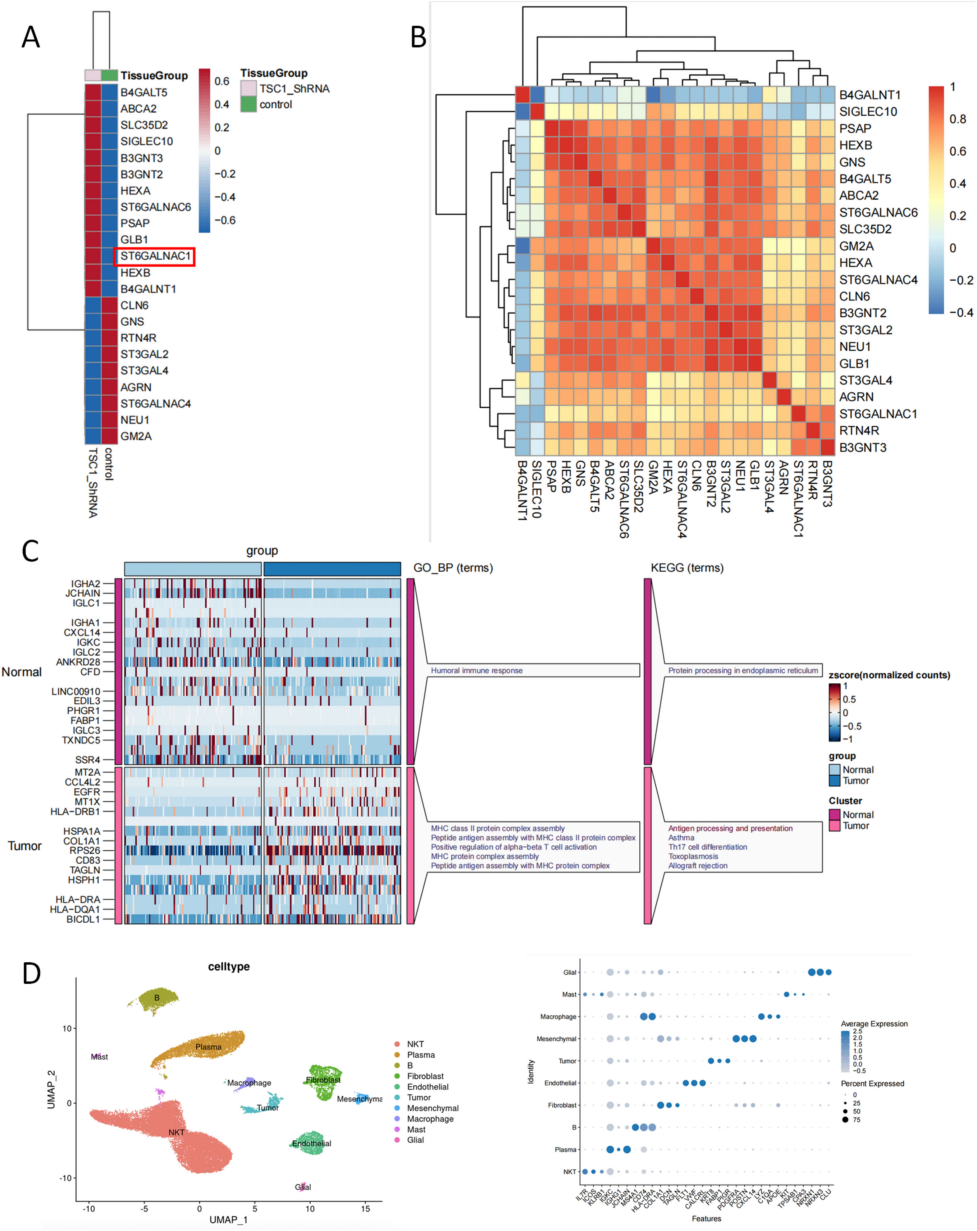
Comprehensive analysis of sialic acid-related gene expression and its association with phenotypic characteristics at single-cell levels. **(A)** Expression patterns of sialic acid-related gene sets and their correlation with phenotypic features. **(B)** Correlation analysis of sialic acid genes and identification of potential therapeutic targets. **(C)** Multi-omics visualization of gene expression and functional enrichment analysis in normal and tumor groups. **(D)** Identification of major cell types and their marker gene expression. (left) UMAP plot showing cell-type clustering. (right) Dot plot showing marker gene expression across clusters.

This study systematically analyzed the expression patterns of sialic acid-related genes and their relationships with phenotypic characteristics using transcriptomic and single-cell sequencing data. At the transcriptomic level (**Figure 3A**), heatmap analysis showed that ST6GALNAC1 was significantly upregulated in the TSC1_ShRNA knockdown cell line compared to the control group. This result is consistent with previous studies and suggests that this gene plays a key role in the regulation of cell surface glycosylation, potentially affecting immune recognition and tumor immune evasion. ST6GALNAC1 has been shown to catalyze the addition of sialic acid to glycoproteins and glycolipids, a process that impacts cell signaling, cell adhesion, and immune cell interactions, particularly in tumor tissues (26–28). The overexpression of this gene in tumor samples may represent a mechanism by which tumor cells escape immune surveillance, marking a sign of tumor progression.

Moreover, genes such as B4GALNT5 and ABCA2 also showed higher expression patterns, which may be closely related to intracellular material transport and metabolic processes, processes that are crucial for maintaining the tumor-supportive microenvironment. The correlation between these genes and ST6GALNAC1 suggests that they may cooperate within a synergistic network to promote cancer cell survival or migration, which is consistent with previous studies on the synergistic role of sialic acid transferases and other glycosylation-related enzymes in cancer (29–32).

In the gene correlation analysis (**Figure 3B**), clustering results for genes such as B4GALNT1, SIGLEC10, and PSAP further support the idea that these genes may participate in similar biological processes or pathways, particularly those related to inflammation, immune regulation, and tumor progression. SIGLEC10, an sialic acid-binding immunoglobulin-like lectin, is closely related to immune cell function, suggesting that sialic acid-modified glycoproteins may play a role in immune evasion and inflammation regulation within the tumor microenvironment (33–35).

In the functional enrichment analysis (**Figure 3C**), we found that gene pathways differed significantly between the normal and tumor groups. Genes in the normal group were mainly enriched in immune response-related pathways, such as humoral immune response, while genes in the tumor group were primarily involved in processes such as MHC class II protein complex assembly, antigen processing, and presentation. KEGG analysis further showed that the tumor group was closely related to immune-related pathways such as antigen processing and presentation, and asthma, suggesting that sialic acid-related genes may influence the tumor microenvironment through immune regulation mechanisms.

To delineate the cellular heterogeneity within the analyzed tissue, we performed unsupervised clustering and UMAP projection. This analysis identified ten transcriptionally distinct cell populations, including NKT cells, plasma cells, B cells, fibroblasts, endothelial cells, tumor cells, mesenchymal cells, macrophages, mast cells, and glial cells (**Figure 3D**, left). The spatial separation of clusters in the UMAP plot indicates clear transcriptional diversity among these populations, reflecting the complex multicellular architecture of the tumor microenvironment. To further define the molecular identity of each cluster, we examined the expression of canonical marker genes across cell types using a dot plot visualization (**Figure 3D**, right). Distinct expression patterns were observed: IL7R and ICOS were enriched in the NKT cluster, IGKC and IGHG1 were highly expressed in B and plasma cells, while COL1A1 and DCN were specifically upregulated in fibroblasts. Endothelial cells displayed high levels of VWF and CLDN5, and macrophages were characterized by strong expression of LYZ and APOE. Notably, glial cells exhibited specific expression of NRXN1 and CLU, confirming their neural identity. These findings collectively demonstrate a heterogeneous cellular ecosystem comprising immune, stromal, and tumor components. Together, these results validate the accuracy of cell-type annotation and highlight the complex immune–stromal interactions that define the tumor microenvironment landscape.

Single-cell RNA sequencing revealed 11 transcriptionally distinct clusters (0–10) within colorectal tumor samples (**Supplementary Figure S1A**). UMAP visualization and marker gene analysis (**Supplementary Figure S1B**) confirmed clear segregation of epithelial, immune, stromal, and endothelial populations, validating accurate cell-type annotation. Functional enrichment of differentially expressed genes showed that GO terms were mainly associated with cell adhesion, extracellular matrix organization, and immune regulation, while KEGG pathways were enriched in PI3K–Akt signaling, ECM–receptor interaction, focal adhesion, and complement and coagulation cascades (**Supplementary Figure S2**). These findings indicate that transcriptional heterogeneity among clusters is closely linked to tumor–immune and extracellular matrix remodeling processes.

In conclusion, our research results indicate that sialic acid-related genes, especially ST6GALNAC1, play an important role in immune regulation and tumor progression. Therefore, they may become new therapeutic targets for future cancer treatments. Further studies on how these genes function in the tumor microenvironment and immune evasion may provide new strategies for cancer therapy.

### 3.4 TSC1 deficiency enhances proliferation and migration of colon cancer cells

**Figure 4:**
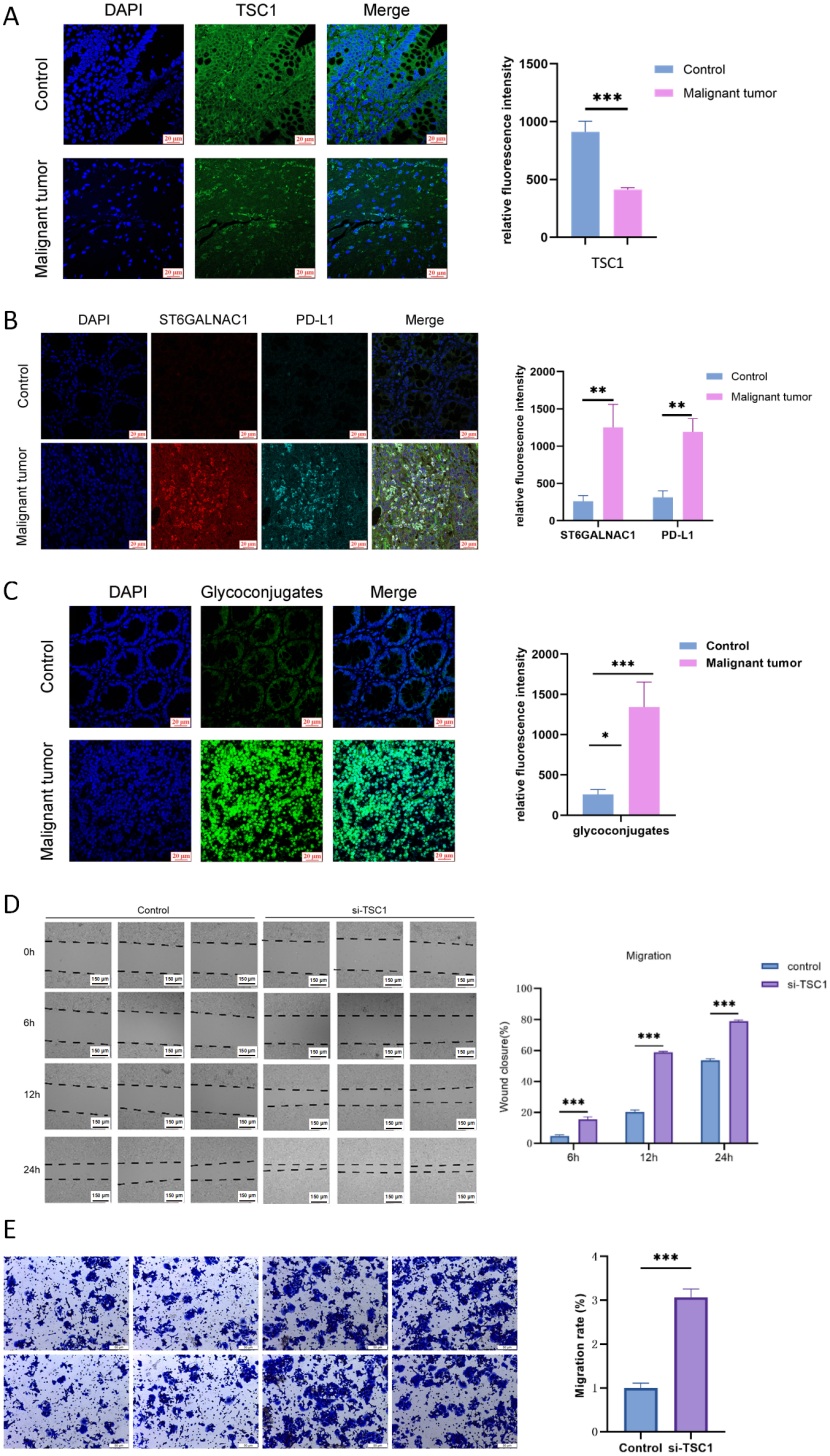
TSC1 deficiency promotes proliferation and migration of colon cancer cells. **(A)** Immunofluorescence staining of TSC1 in normal and cancerous tissues. **(B)** Immunofluorescence staining of ST6GALNAC1 and PD-L1 in normal and cancerous tissues. **(C)** Analysis of sialylation patterns across normal mucosa, adenomatous polyps, and malignant tissues. **(D)** Wound healing assay to assess cell proliferation after TSC1 knockdown. **(E)** Representative images of crystal violet-stained migrated cells. Data are presented as mean ± SEM (n=3). ***p < 0.001, **p < 0.01, *p < 0.05 vs control group.

Immunofluorescence analysis revealed decreased TSC1 expression alongside elevated levels of ST6GALNAC1 and PD-L1 in colorectal cancer tissues compared to adjacent normal tissues (**Supplementary Figures S4A, B**). PD-L1 upregulation appears mediated through dual mechanisms: transcriptional induction via IFN-γ/STAT1 signaling activated by chronic inflammation/genomic instability, and post-translational stabilization through ST6GALNAC1-mediated sialylation that enhances PD-1 binding affinity and membrane retention, establishing a positive feedback loop. Further analysis revealed that the sialylation level in cancer cells was significantly higher than that in normal cells (**Figure 4C**), suggesting that sialylation may play a critical role in malignant transformation. Functional assays confirmed that TSC1 knockdown significantly enhanced tumor cell proliferation and migration. Scratch wound healing assays showed accelerated closure rates in TSC1-deficient groups versus controls (**Figure 4D**), attributable to mTORC1 pathway dysregulation: TSC1 depletion releases Rheb-GTPase inhibition, activating mTORC1 downstream effectors S6K1/4E-BP1 to drive ribosome biogenesis/translation, while metabolic reprogramming provides proliferative energy support. Transwell invasion assays further validated enhanced migration in TSC1-knockdown cells (**Figure 4E**), mediated through: (1) mTORC1/S6K1-dependent cytoskeletal remodeling via FAK/Paxillin/Cofilin; (2) HIF-1α/c-Myc-induced metabolic reprogramming and MMP-2/MMP-9 secretion; and (3) potential autophagy/integrin signaling modulation. These findings collectively establish TSC1 deficiency as a multi-faceted driver of colorectal cancer progression through mTORC1 hyperactivation and sialylation-dependent immune evasion.

### 3.5 TSC1–mTORC1 signaling enhances immune evasion by regulating PD-L1 sialylation in colorectal cancer

**Figure 5:**
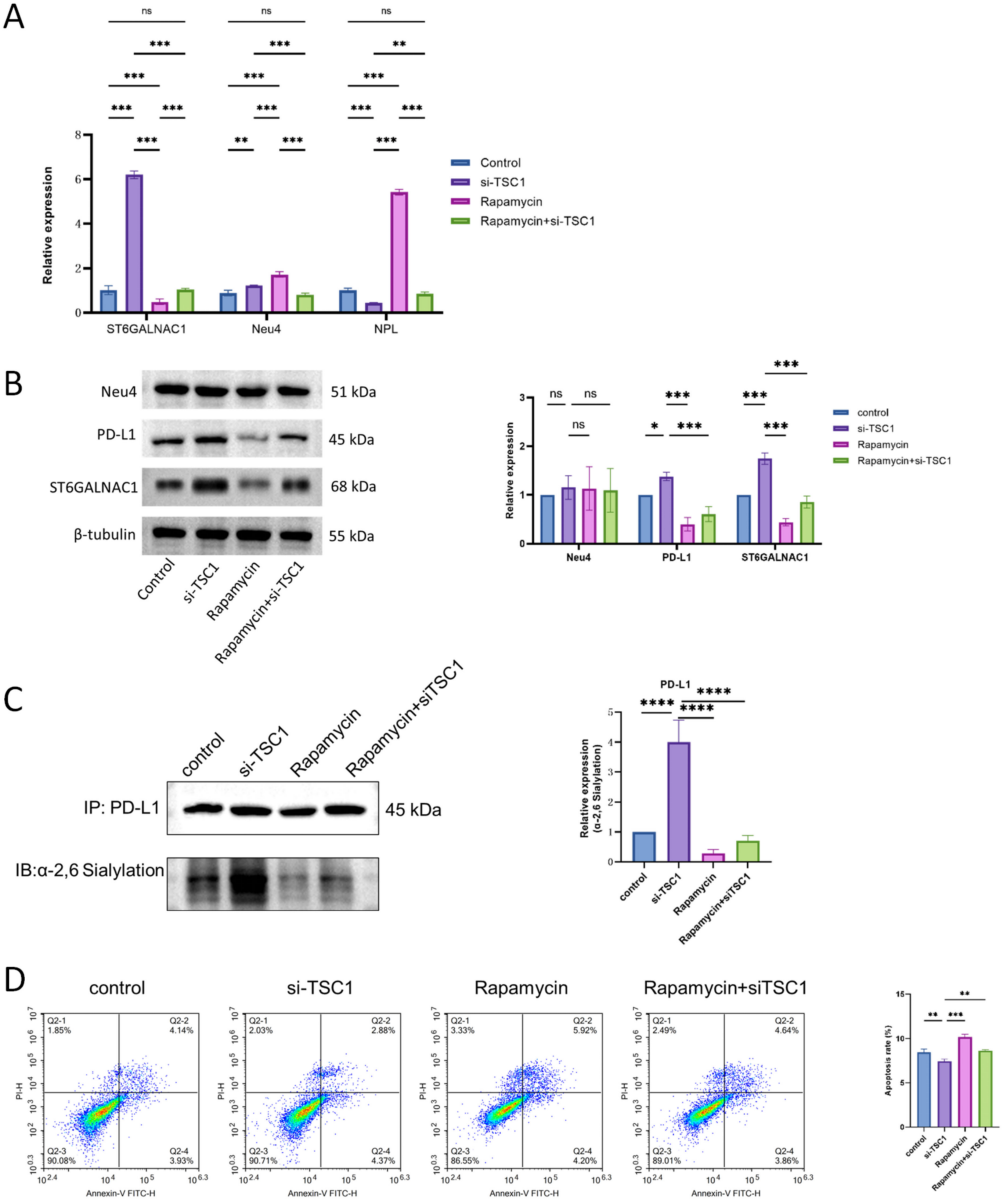
TSC1 deficiency remodels sialic acid metabolic homeostasis via the mTORC1-S6K1 signaling pathway and promotes tumor immune escape. **(A)** qPCR analysis of ST6GALNAC1, Neu4, and NPL mRNA expression. **(B)** Representative Western blot images showing the expression levels of ST6GALNAC1, Neu4, and PD-L1 in indicated groups. **(C)** Analysis of PD-L1 sialylation levels by lectin blot assay. **(D)** Assessment of CD8+ T cell cytotoxic function. Data are presented as mean ± SEM (n=3). ***p < 0.001, **p < 0.01, *p < 0.05 vs control group.

qRT-PCR analysis revealed that ST6GALNAC1 expression was markedly upregulated following TSC1 deletion, whereas the expression of the desialylating enzyme NEU4 showed a slight increase, and NPL expression was significantly reduced (**Figure 5A**). In contrast, treatment with rapamycin markedly decreased ST6GALNAC1 expression while significantly elevating the mRNA levels of NEU4 and NPL. Furthermore, rescue experiments confirmed that TSC1 knockdown effectively reversed the regulatory effects of rapamycin on these genes. Western blot analysis further validated efficient TSC1 knockdown and its associated molecular effects (**Figure 5B**).

Lectin blot analysis using Sambucus nigra agglutinin (SNA) revealed that the α2,6-sialylation level was markedly elevated in TSC1-deficient cells, whereas rapamycin treatment significantly reduced α2,6-sialylation (**Figure 5C**). Notably, rapamycin administration in TSC1-knockdown cells effectively reversed the enhanced α2,6-sialylation induced by TSC1 loss.

Functionally, flow cytometric analysis showed that both early and late apoptosis rates were significantly reduced in TSC1-deficient cells, while restoration of TSC1 expression markedly increased apoptotic levels (**Figure 5D**).

Collectively, these results indicate that the loss of TSC1 enhances cellular α2,6-sialylation, thereby suppressing apoptosis and promoting tumor cell survival, suggesting that TSC1-mediated regulation of sialylation plays a critical role in malignant progression.

### 3.6 TSC1 deficiency induces an immunosuppressive microenvironment and reduces the efficacy of PD-1 blockade in colorectal cancer

**Figure 6:**
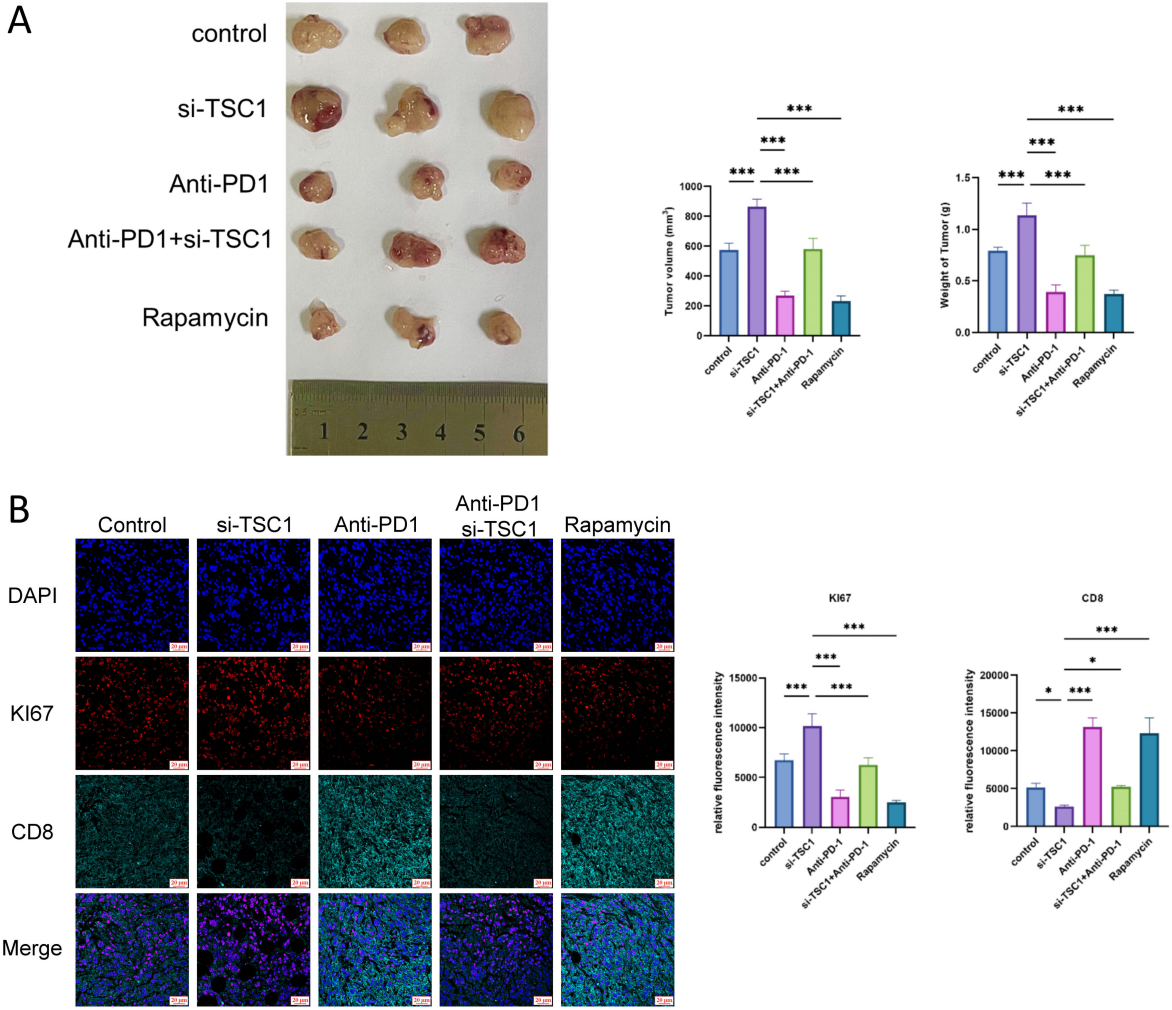
Animal experiments validate the immunosuppressive microenvironment after TSC1 knockdown. **(A)** Tumor volume and weight in the TSC1 knockdown model treated with anti-PD-1 therapy. **(B)** TSC1 knockdown MC38 cell subcutaneous xenograft model. Data are presented as mean ± SEM (n=3). ***p < 0.001, *p < 0.05 vs control group.

This study systematically evaluated the impact of TSC1 deficiency on tumor proliferation, immune microenvironment, and therapeutic responses. *In vivo* experiments further confirmed that TSC1 knockdown significantly promoted tumor growth (with TSC1-deficient groups showing maximal tumor weight and volume), while anti-PD-1 monotherapy and rapamycin exhibited the most potent tumor-suppressive effects (**Figure 6A**). In TSC1-knockdown groups, tumor cells exhibited significantly higher fluorescence intensity of proliferation marker KI67 compared to controls, while anti-PD-1 monotherapy and rapamycin treatment groups showed the lowest KI67 levels (**Figure 6B**). CD8^+^ T cell infiltration increased in both TSC1-knockdown and anti–PD-1 monotherapy groups. In TSC1-deficient tumors, however, infiltrating CD8^+^ T cells displayed an exhausted phenotype, consistent with PD-L1 sialylation–driven immunosuppression. Combination therapy produced intermediate tumor proliferation and CD8^+^ T cell infiltration, suggesting partial but incomplete reversal of TSC1 deficiency–induced immune evasion, likely constrained by additional immunosuppressive pathways such as IDO1 induction or Treg recruitment.

Mechanistic studies showed that TSC1 deficiency contributes to immunotherapy resistance through mTORC1-dependent pathways, including enhanced PD-L1 sialylation, and mTORC1-independent mechanisms, such as Treg infiltration. Rapamycin achieved superior efficacy, associated with both suppression of tumor cell proliferation and improved T cell metabolic fitness. These results support combined targeting of mTORC1 signaling and immune checkpoints to modulate the tumor microenvironment in TSC1-deficient malignancies.

## 4 Discussion

The present study provides compelling evidence that TSC1 serves as a crucial molecular nexus linking mTORC1 signaling, sialylation metabolism, and immune regulation in colorectal cancer. Our findings significantly expand the current understanding of TSC1’s biological functions beyond its canonical role as an mTORC1 inhibitor, revealing its novel capacity to modulate tumor immune evasion through glycosylation-dependent mechanisms.

The correlation analyses establish TSC1 as a robust prognostic biomarker in CRC, with its decreased expression associated with advanced disease stages and poorer survival outcomes. These observations align with previous reports of TSC1’s tumor suppressive functions in other malignancies, while uniquely extending its clinical relevance to the context of CRC immune microenvironment regulation (36–40). Notably, our data demonstrate that TSC1 expression levels show significant inverse correlations with established markers of immune evasion, suggesting its potential utility in predicting response to immunotherapy.

At the mechanistic level, we identified a previously underappreciated regulatory axis through which TSC1 restrains PD-L1 sialylation by coordinately regulating the expression of the sialylation-related enzymes ST6GALNAC1 and NEU4. Under physiological conditions, TSC1 suppresses mTORC1 activity to maintain glycosylation homeostasis. In contrast, loss of TSC1 results in mTORC1 hyperactivation, leading to upregulation of ST6GALNAC1 and downregulation of NEU4, thereby enhancing the α2,6-linked sialylation of PD-L1. This modification increases PD-L1 stability and its interaction with PD-1, ultimately facilitating tumor immune evasion. This TSC1–mTORC1–sialylation axis provides a mechanistic explanation for how TSC1 influences the tumor immune microenvironment through glycosylation control. Moreover, the strong inverse relationship between TSC1 expression and sialylation-associated immune suppression suggests that TSC1 expression levels may serve as a biomarker linking metabolic regulation to immune checkpoint functionality, offering a conceptual basis for future studies on immunotherapy response prediction.

To expand this model, we further examined NPL, a key enzyme that catabolizes free sialic acids and thereby regulates the intracellular substrate pool for glycosylation. Together with NEU4, which removes terminal sialic acids from glycoconjugates, NPL provides an additional layer of control over cellular sialylation homeostasis. Both enzymes have been implicated in tumor progression, and our findings confirm that their downregulation correlates with the immunosuppressive phenotype of TSC1-deficient CRC cells. Integrating NEU4 and NPL into the TSC1–mTORC1–sialylation axis further refines the mechanistic framework of PD-L1 hypersialylation–driven immune escape, highlighting a coordinated metabolic control of sialic acid turnover in tumor immune regulation.

The functional impact of TSC1-mediated regulation of sialylation was further validated in our *in vivo* colorectal tumor models. TSC1-deficient tumors exhibited marked infiltration of CD8^+^ T cells, yet these lymphocytes displayed clear features of functional exhaustion, consistent with enhanced PD-L1 stability and engagement with PD-1 resulting from altered glycosylation patterns. This immunosuppressive phenotype may underlie the limited responsiveness of TSC1-deficient tumors to PD-1 blockade and suggests that combination strategies co-targeting the PD-1/PD-L1 axis and its glycosylation-dependent regulation may achieve superior therapeutic outcomes. Our therapeutic experiments with rapamycin revealed several important findings with both mechanistic and clinical significance. First, mTOR inhibition appears to partially reverse the sialylation imbalance caused by TSC1 deficiency, supporting the central role of this pathway in glycosylation regulation. This observation underscores the pivotal position of the TSC1/mTOR pathway in regulating immune escape. More importantly, although rapamycin is traditionally considered a broad immunosuppressant, we observed in this study that it enhances CD8^+^ T cell function and suppresses tumor growth. This “apparent paradox” stems from the context-specific role of mTOR signaling in different immune environments. In the tumor microenvironment, characterized by chronic antigen stimulation and high metabolic pressure, CD8^+^ T cells often exhibit hyperactivated mTORC1 signaling, metabolic dysregulation, and terminal exhaustion (41–45). Low-dose rapamycin intervention helps alleviate this pathological activation state, restores metabolic homeostasis, and promotes the accumulation of TCF-1^+^ stem-like T cells, thereby enhancing their proliferative potential and cytokine production capacity (46–48). Compared to PD-1 blockade alone, this strategy may simultaneously target PD-L1 sialylation status and immunometabolic regulation, addressing multiple immune escape pathways and synergistically enhancing anti-tumor immunity. Furthermore, under specific conditions, rapamycin can also impair the function of immunosuppressive cells in the tumor microenvironment. For example, studies have shown that rapamycin can reduce the immunosuppressive activity of regulatory T cells (Tregs) without significantly affecting their numbers, an effect potentially linked to the downregulation of Akt signaling (49). Additionally, rapamycin inhibits the polarization of tumor-associated macrophages (TAMs) toward the pro-tumor M2 phenotype (50–52). These regulatory effects help alleviate the immunosuppressive state in the tumor microenvironment, creating more favorable conditions for the effector activation and functional infiltration of CD8^+^ T cells. Our results suggest that in the context of TSC1 deficiency-driven metabolic abnormalities and glycosylation dysregulation, mTOR inhibition does not manifest as systemic immunosuppression in the traditional sense but rather acts as a selective reprogramming strategy for fine-tuning the immune microenvironment of the TME. This discovery has clear clinical translational value, particularly in colorectal cancer patients with TSC1 pathway abnormalities, where combining mTOR inhibitors may enhance the sensitivity to immune checkpoint therapy and expand the applicability of precision immunotherapy.

Our study has several limitations that warrant further reflection and refinement. First, although we primarily focused on the α2,6-sialylation process mediated by ST6GALNAC1, other sialyltransferases and glycosylation modifications may also contribute to the observed phenotypes. Therefore, future studies should expand to include a broader range of glycosyltransferase families to comprehensively elucidate the relationship between glycosylation networks and immune regulation. Second, our detection of sialylation mainly relied on SNA lectin blotting, which lacks the precision to resolve site-specific modifications on particular proteins. To gain a deeper understanding of the glycosylation characteristics of PD-L1, future work could integrate glycoproteomic analysis via mass spectrometry, enabling site-specific and absolute quantitative analysis of glycosylation patterns on key immune proteins, thereby enhancing the resolution and credibility of mechanistic investigations. Third, in terms of mechanistic validation, although we established the regulatory axis of TSC1–mTORC1–ST6GALNAC1/NEU4 through functional and expression data, key direct evidence is still lacking. To verify the specificity and directness of this pathway, we plan to employ chromatin immunoprecipitation (ChIP) assays in follow-up studies to further determine whether S6K1 or its downstream transcription factors directly bind to the promoter regions of target genes. Our *in vivo* model cannot fully recapitulate the complexity of human tumor-immune interactions, particularly the heterogeneity of immune cell populations in clinical settings. Future studies that incorporate patient-derived tissue cultures and humanized mouse models, combined with spatially resolved methods such as IHC and multiplex immunofluorescence, would enable systematic clinical validation of TSC1 expression status and its relationship with PD-L1 glycosylation, thereby advancing this research toward higher translational applicability.

From a translational perspective, our findings suggest several promising therapeutic strategies. Pharmacological modulation of sialylation enzymes, particularly ST6GALNAC1 inhibitors, might synergize with existing immunotherapies in TSC1-deficient CRCs. Additionally, metabolic interventions targeting the mTOR-sialic acid axis could provide novel approaches to overcome immune checkpoint resistance. The development of clinical-grade sialylation modulators and the identification of predictive biomarkers for this approach represent important directions for future research.

In conclusion, this study identifies TSC1 as a key regulator of tumor-associated sialylation and immune evasion in colorectal cancer. By elucidating the molecular links between mTORC1 signaling, glycosylation metabolism, and immune checkpoint regulation, our work provides a mechanistic basis for developing more effective therapeutic strategies for immune-resistant colorectal cancer. These findings may also have broader implications for understanding and targeting glycosylation-dependent immune evasion mechanisms across multiple cancer types.

## Data Availability

The datasets presented in this study can be found in online repositories. The names of the repository/repositories and accession number(s) can be found in the article/**Supplementary Material**.
