# Supplementary material for: TSC1 deficiency drives immune evasion in colorectal cancer via mTORC1-mediated dysregulation of PD-L1 sialylation
Source: Front Immunol. 2025 Dec 9;16:1692210. doi: 10.3389/fimmu.2025.1692210 (PMC12723005; doi:10.3389/fimmu.2025.1692210)
Supplement: Supplementary file 1 [file Table1.docx]

Table S1. Association of TSC1 Expression with Immune Cell Infiltration Patterns Using Pearson Correlation.

|  | p | r |
| --- | --- | --- |
| NK.cells.resting | 2.99E-05 | 0.18335823 |
| Eosinophils | 0.002237024 | 0.134805756 |
| T.cells.CD8 | 0.009730457 | -0.11415888 |
| Monocytes | 0.040696844 | 0.090483586 |

Table S2. Primer sequences for qRT-PCR.

| Gene Name | Primer Sequence (5' to 3') |
| --- | --- |
| ****TSC1**** | Forward:CTGGACAGACTGATACAGCAGG Reverse:TGCGGATCTCATCTGAAGGAGG |
| ****NEU4**** | Forward:ACCGCCGAGAGTGTTTTGG Reverse:CGTGGTCATCGCTGTAGAAGG |
| ****ST6GALNAC1**** | Forward:CGAAATAGGAGGCCTTCAGA Reverse:AGAGAGTGAGGTTGGGCAGA |
| ****NPL**** | Forward:GTGGGTGACAAAAGGGAAGGAC Reverse:GCAATGACAGCGATGCCATCAG |
| ****GAPDH**** | Forward:GTCTCCTCTGACTTCAACAGCG  Reverse:ACCACCCTGTTGCTGTAGCCAA |

Table S3. TSC1 shRNA sequences used for lentiviral knockdown

| **Name** | **Target Sequence (5'→3')** |
| --- | --- |
| shTSC1-1 | CCGGG**CTCAGAGATCGGACTTATT**TCTCGAG**AAATAAGTCCGATCTCTGAGC**TTTTTG |
| shTSC1-2 | CCGGG**CTTGGTCTGAGAGATATAT**TCTCGAG**AATATATCTCTCAGACCAAGC**TTTTTG |
| shCtrl | CCGG**CAACAAGATGAAGAGCACCA**ACTCGAG**TTGGTGCTCTTCATCTTGTTG**TTTTTG |

**Supplementary Figure 1. Single-cell transcriptomic analysis identifies major cell clusters and their marker gene expression patterns.**(A) UMAP visualization showing 11 transcriptionally distinct clusters (0–10) identified from single-cell RNA sequencing data. Each dot represents a single cell, and colors indicate different clusters. (B) Dot plot displaying representative marker gene expression across identified clusters. Dot color indicates the average expression level, and dot size represents the percentage of cells expressing the corresponding gene. Distinct expression signatures across clusters support accurate cell-type annotation.

**Supplementary Figure 2. GO and KEGG enrichment analyses of differentially expressed genes (DEGs).** (A) GO enrichment analysis showing significantly enriched terms across three main categories: biological process (BP), cellular component (CC), and molecular function (MF). The x-axis represents the number of enriched genes, and the color scale indicates adjusted p-values (p.adjust). (B) KEGG pathway enrichment analysis revealing that DEGs are significantly associated with the PI3K-Akt signaling pathway, ECM-receptor interaction, estrogen signaling pathway, focal adhesion, and immune-related pathways such as complement and coagulation cascades. The color gradient reflects the adjusted significance level, with red indicating higher enrichment significance. These results suggest that DEGs are primarily involved in cell adhesion, extracellular matrix remodeling, and immune regulation.
